# Synthesis, Self-Assembly, and Drug-Release Properties of New Amphipathic Liquid Crystal Polycarbonates

**DOI:** 10.3390/nano8040195

**Published:** 2018-03-27

**Authors:** Yujiao Xie, Xiaofeng Liu, Zhuang Hu, Zhipeng Hou, Zhihao Guo, Zhangpei Chen, Jianshe Hu, Liqun Yang

**Affiliations:** 1 Center for Molecular Science and Engineering, College of Science, Northeastern University, Shenyang 110819, China; xieyujiao5819573@gmail.com (Y.X.); 1510048@stu.neu.edu.cn (X.L.); 1710059@stu.neu.edu.cn (Z.H.); 1670175@stu.neu.edu.cn (Z.H.); 1610046@stu.neu.edu.cn (Z.G.); chenzhangpei@mail.neu.edu.cn (Z.C.); 2 Key Laboratory of Reproductive Health, Liaoning Research Institute of Family Planning, Shenyang 110031, China

**Keywords:** amphipathic polycarbonates, cholesteryl, liquid crystal, self-assembly, drug release

## Abstract

New amphiphilic liquid crystal (LC) polycarbonate block copolymers containing side-chain cholesteryl units were synthesized. Their structure, thermal stability, and LC phase behavior were characterized with Fourier transform infrared (FT-IR) spectrum, ^1^H NMR, gel permeation chromatographic (GPC), thermogravimetric analysis (TGA), differential scanning calorimetry (DSC), polarizing optical microscope (POM), and XRD methods. The results demonstrated that the LC copolymers showed a double molecular arrangement of a smectic A phase at room temperature. With the elevating of LC unit content in such LC copolymers, the corresponding properties including decomposition temperature (*T*_d_), glass temperature (*T*_g_), and isotropic temperature (*T*_i_) increased. The LC copolymers showed pH-responsive self-assembly behavior under the weakly acidic condition, and with more side-chain LC units, the self-assembly process was faster, and the formed particle size was smaller. It indicated that the self-assembly driving force was derived from the orientational ability of LC. The particle size and morphologies of self-assembled microspheres loaded with doxorubicin (DOX), together with drug release tracking, were evaluated by dynamic light scattering (DLS), SEM, and UV–vis spectroscopy. The results showed that DOX could be quickly released in a weakly acidic environment due to the pH response of the self-assembled microspheres. This would offer a new strategy for drug delivery in clinic applications.

## 1. Introduction

Biodegradable aliphatic polyesters have received more and more attention in recent years due to their good biodegradability and biocompatibility [1,2]. Among them, four class of polyesters, namely polyglycolide (PGA), poly(1,3-trimethylene carbonate) (PTMC), polylactide (PLA), poly(caprolactone) (PCL), and their copolymers, are the most widely studied and utilized in biomedical fields. However, exploration of their applications has been hampered by several limitations owing to their specific properties, for example, the PGA degradation rate is too fast with respect to the required application time of materials [3,4,5]; and PLA can produce acid degradation products which may cause aseptic inflammation [6]. Although there is no acid degradation product during the degradation of PTMC [7], it lacks functional groups to link bioactive primitives. Therefore, 5-benzyloxy-trimethylene carbonate (BTMC) had been selected as the cyclic monomer to form polycarbonate copolymers containing hydroxyl groups [8,9,10].

It has been shown that various sensory mechanisms of organisms are related to the self-assembly behavior of liquid crystals (LCs) in vivo [11]. Another important point is that LCs can self-assemble to form a variety of ordered structures which are responsive to temperature, pH, stress, magnetic field, and other external conditions [12,13,14]; as a result, LC structures present considerable advantages in the transport and delivery of diverse active molecules and drugs [15]. Cholesterol is an indispensable material in the animal and human body, and its derivatives were found to have LC properties at earlier time [16,17,18]. Therefore, as a natural biomesogen, cholesterol has become an attractive candidate for forming new smart, responsive, and biodegradable LC polymer materials. Nowadays, it has been reported that the cholesteryl groups could be employed as mesogenic units to fabricate side-chain [19,20,21,22,23] and main-chain LC polymers [24,25,26,27,28]. However, there is limited research on aliphatic polycarbonates with side-chain cholesteryl LC units [21,29].

The use of polymers for drug delivery is one of the most important drug delivery systems, and the drug-loaded nanoparticle formed with polymers are faster and more convenient in medical treatment since they can be injected to avoid major surgery [4,30,31]. The nanoparticles with tunable properties may be combined by the self-assembly of LC groups and amphiphiles of appropriate chemical structures, such as PEGylated polycarbonates [32,33]. Furthermore, LC polymers, created by the principles of self-assembly and nanocarriers, have been studied with the incorporation of different bioactive macromolecules such as siRNA, plasmid DNA, peptides, and proteins [34,35,36,37,38]. However, there is rarely research on the self-assembly of LC polymers with cholesterol [14]. Thus, it is very significant to the scientific community to study biodegradable aliphatic polycarbonates with cholesteryl LC groups, and their potential medical and clinical application value.

In the previous work, we reported the synthesis and self-assembled morphology of new side-chain diosgenin-functionalized block copolymers with an aliphatic polycarbonate backbone [39]. In this study, a series of new cholesterol-functionalized amphipathic LC copolymers based on aliphatic polycarbonates were synthesized. The design aim is for the hydrophilic segment to be introduced into the aliphatic polycarbonate to improve the hydrophilicity of the copolymer, and the hydroxyl groups are introduced to create reactive sites which can link cholesteryl LC units. The synthesis, thermal stability, and phase behavior of the obtained LC copolymers were characterized by FT-IR, ^1^H NMR, gel permeation chromatographic (GPC), thermogravimetric analysis (TGA), differential scanning calorimetry (DSC), polarizing optical microscope (POM), and X-ray diffraction (XRD) measurements. Subsequently, the pH-responsive self-assembly process of the target copolymers was studied by UV–vis, and their particle size and morphologies were characterized by dynamic light scattering (DLS) and scanning electron microscope (SEM), respectively. Doxorubicin (DOX), a broad-spectrum anticancer drug, was chosen as the drug model to prepare drug-loaded LC copolymer microspheres via a dialysis method. The particle size and morphologies of DOX-loaded microspheres were detected by DLS and SEM, respectively. The drug-loading efficiency and pH-responsive drug release behavior were monitored by UV–vis. It could serve as a potential biomedical material as well as a drug delivery carrier for tumor therapy.

## 2. Experimental Method

### 2.1. Materials

All chemicals were obtained from the indicated sources. 1,3-Trimethylene carbonate (TMC) was purchased from Daigang Biomaterial Co. Ltd. (Jinan, China), and recrystallized twice with acetic ether and then dried 24 h in a vacuum before polymerization. Stannous octanoate (Sn(Oct)_2_) and methoxypolyethylene glycols (mPEG_43_) were purchased from Aldrich and used without purification. Doxorubicin hydrochloride (DOX·HCl) was purchased from Ark Pharm, Inc. (Arlington Heights, IL, USA) Triethylamine (TEA) was purchased from Sinopharm Chemical Reagent Co., Ltd. (Shenyang, China). Tetrahydrofuran (THF) and toluene were dried by treatment with Na and distilled before use. All other solvents and reagents used were purified by standard methods.

### 2.2. Measurements

*FT-IR spectra*. FT-IR spectra were obtained using a PerkinElmer spectrum One (B) spectrometer (PerkinElmer, Foster City, CA, USA). Solid samples were pressed into KBr pellets and liquid samples were drop-casted on KBr pellets.

*^1^H NMR spectra*. ^1^H NMR spectra were obtained using a Bruker ARX 600 (Karlsruhe, Germany) high-resolution NMR spectrometer, and chemical shifts were reported in ppm with tetramethylsilane (TMS) as an internal standard.

*Gel permeation chromatographic (GPC)*. GPC were carried out at room temperature on a Waters 1515 instrument (Shanghai, China) calibrated with THF as an eluent and polystyrene as the standard.

*Thermogravimetric analysis (TGA)*. The thermal decomposition temperature was measured under a nitrogen atmosphere with a Netzsch 209C thermogravimetric analysis (Hanau, Germany) at a heating rate of 20 °C/min.

*Differential scanning calorimetry (DSC)*. The phase transition temperature was determined with a Netzsch 204 (Netzsch, Hanau, Germany) DSC equipped with a cooling system at a heating and cooling rate of 10 °C/min in a nitrogen atmosphere.

*Polarizing optical microscope (POM)*. The optical textures were observed with a Leica DMRX (Leica, Wetzlar, Germany) POM equipped with a Linkam THMSE-600 (Linkam, London, UK) cool and hot stage.

*X-ray diffraction (XRD)*. XRD measurements were performed with nickel-filtered Cu-Kα radiation (λ = 1.54 Å) with a Bruker D8 Advance (Karlsruhe, Germany) powder diffractometer. The temperature-dependent X-ray measurements were carried out in the heating process.

*Dynamic light scattering (DLS)*. The particle size and size distribution of the micelles were determined with DLS using a Zetasizer Nano S instrument (Malvern Instruments Ltd., Worcestershire, UK) with a He–Ne laser (633 nm) set at 173° for the scattering angle at 25 °C.

*Scanning electron microscope (SEM)*. The morphology of the micelles was examined using a Hitachi X650 (Tokyo, Japan). The SEM samples were prepared by depositing several drops of the samples suspension onto the surface of cleaned aluminum foil, and the samples were quenched by liquid nitrogen, then freeze-dried in a vacuum at −50 °C for 24 h. The samples were coated with a thin film of gold before measuring.

*UV–vis.* Light transmittance test and drug absorbance were measured using a TU-1901 double-beam UV–visible spectrophotometer (Beijing Purkinje General Instrument Co., Ltd., Beijing, China).

### 2.3. Synthesis of the Block Copolymers

**Scheme 1:**
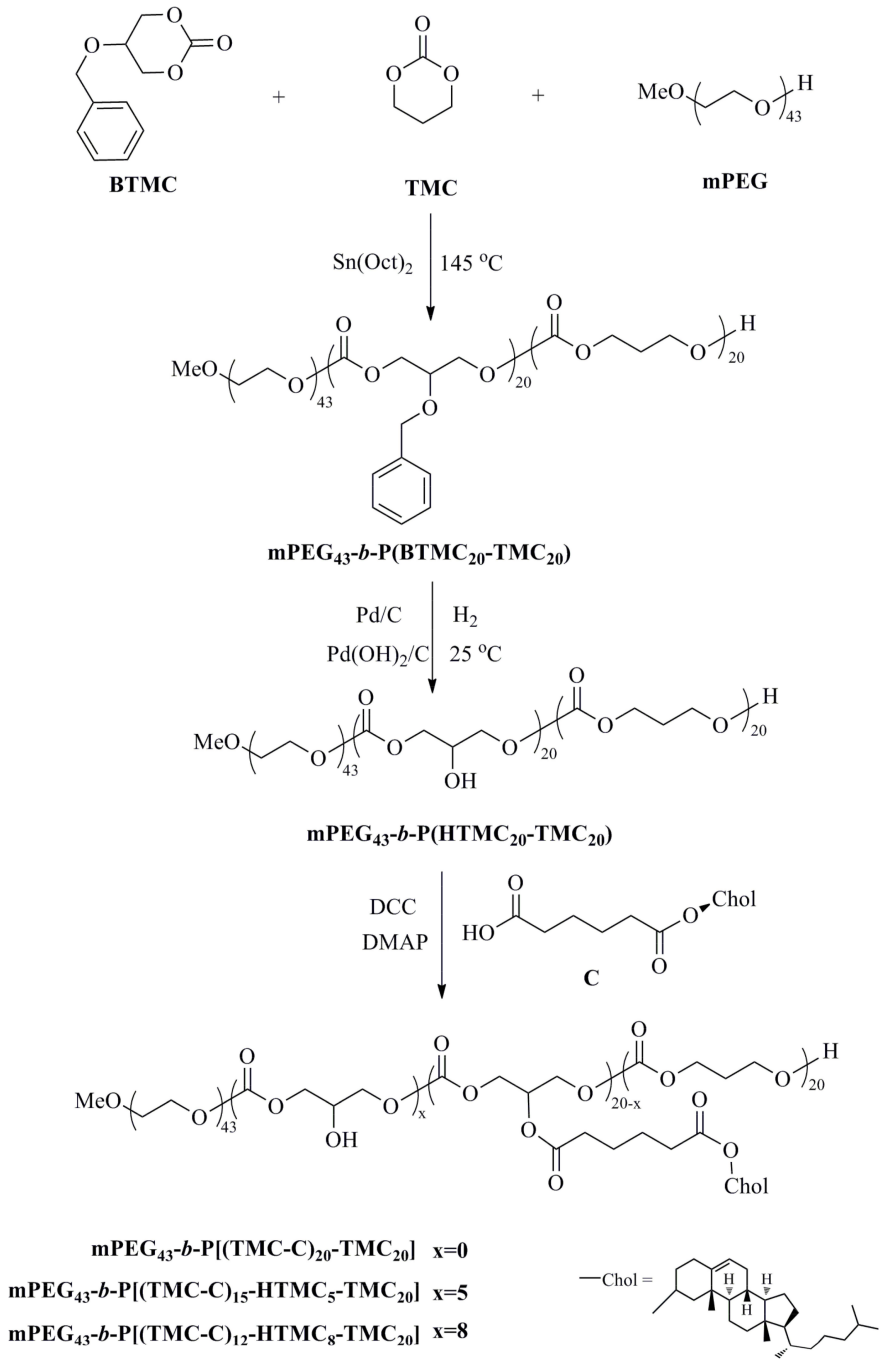
Synthetic route of the copolymers (C: 6-cholesteroxy-6-oxocaproic acid).

The synthetic route of the block copolymers is outlined in Scheme 1. The cyclic monomer BTMC and the chiral LC monomer 6-cholesteroxy-6-oxocaproic acid (C) were synthesized according to our previous works [6,29].

#### 2.3.1. Synthesis of mPEG_43_-*b*-P(BTMC_20_-TMC_20_)

BTMC (10.4 g, 0.05 mol), TMC (4.9 g, 0.05 mol), and mPEG (3.8 g, 0.002 mol) were placed in a polymerization flask, and then Sn(Oct)_2_ toluene solution (0.13 mol/L, 1.6 mL) as a catalyst was added to the above flask. The flask was sealed in a vacuum and placed in an oil bath kept at 145 °C. The reaction was maintained for 24 h. After the completion of polymerization, the crude product was purified by dissolving it in dichloromethane and precipitated in methanol, and then dried in a vacuum until the sample mass was constant.

IR (KBr, cm^−1^): 2961, 2875 (−CH_2_–, –CH_3_); 1752 (C=O); 1531, 1455 (–Ph); 1241 (C–O–C); ^1^H NMR (δ, CDCl_3_, 600 MHz): δ = 7.26 (Ph–***H***), δ = 4.65 (Ph–C***H***_2_O–), δ = 4.25 (–OC***H***_2_–CH–C***H***_2_O), δ = 3.85 (–OCH_2_–C***H***–CH_2_O– in PBTMC), δ = 3.64 (–OC***H***_2_– in mPEG), δ = 2.03 (–OCH_2_–C***H***_2_–OCH_2_– in PTMC).

#### 2.3.2. Synthesis of mPEG_43_-*b*-P(HTMC_20_-TMC_20_)

mPEG_43_-*b*-P(BTMC_20_-TMC_20_) (2.1 g) was dissolved in methanol/THF (1:1, 100 mL) and placed in a 250 mL three-necked flask with a magnetic stirrer. Pd/C (0.10 g, 5%) and Pd(OH)_2_/C (0.10 g, 5%) were added to the above solution. The reaction mixture was stirred for 48 h in a hydrogen system at 25 °C. After the reaction, Pd/C and Pd(OH)_2_/C were filtered out. The filtrate was evaporated to dryness, and then dried in a vacuum until constant weight was obtained.

IR (KBr, cm^−1^): 3376 (–OH); 2955, 2853 (–CH_2_–, –CH_3_); 1750 (C=O); 1252 (C–O–C); ^1^H NMR (δ, DMSO, 600 MHz): δ = 5.45 (–OH), δ = 4.09 (–OC***H***_2_–CH–C***H***_2_O–), δ = 3.93 (–OCH_2_–C***H***–CH_2_O– in PHTMC), δ = 3.51 (–OC***H***_2_– in mPEG), δ = 1.95 (–OCH_2_–C***H***_2_–OCH_2_– in PTMC).

#### 2.3.3. Synthesis of mPEG_43_-*b*-P[(TMC-C)_20−*x*_-HTMC*_x_*-TMC_20_]

The chiral monomer C (containing a –COOH group) was dissolved in dichloromethane and added to a 250 mL three-necked flask with a magnetic stir bar. Then, *N*,*N*′-dicyclohexylcarbodiimide (DCC) and 4-dimethylaminopyridine (DMAP) were dissolved in dichloromethane, and added dropwise to the mixture. After stirring for 0.5 h, mPEG_43_-*b*-P(HTMC_20_-TMC_20_) (containing –OH groups) dissolved in dichloromethane was added dropwise to the above mixture. The reaction mixture was stirred for 72 h at room temperature. The fabrication of mPEG_43_-*b*-P(HTMC_20_-TMC_20_) and the monomer C is shown in Table 1. The resulting mixture was washed with water and *N*,*N*′-dicyclohexyl urea was precipitated and filtered off. The filtrate was evaporated and concentrated. The crude product was purified by dissolving it in dichloromethane and precipitated in methanol. The precipitate was dried in a vacuum until constant weight was obtained.

IR (KBr, cm^−1^): 2951, 2868(–CH_2_–, –CH_3_); 1745 (C=O); 1674 (C=C); 1256, 1171 (C–O–C); ^1^H NMR (δ, CDCl_3_, 600 MHz): δ = 5.37 (–C***H***=C in cholesteryl), δ = 5.27 (–OCH_2_–C***H***–CH_2_O– in PHTMC), δ = 4.60 (–COOC***H***< in cholesteryl), δ = 4.36−4.25 (–OC***H***_2_–CH–C***H***_2_O), δ = 3.64 (–OC***H***_2_– in mPEG), δ = 2.37−0.68 (the rest of the protons from PTMC, cholesteryl and –COO(C***H_2_***)_4_COO–).

### 2.4. Nanoprecipitation of Copolymers

The three amphiphilic LC block copolymers mPEG_43_-*b*-P[(TMC-C)_20−*x*_-HTMC*_x_*-TMC*_y_*] have unique features, with mPEG as a hydrophilic segment and P[(TMC-C)_20−*x*_-HTMC*_x_*-TMC*_y_*] chains as hydrophobic segments. The micelles were prepared as follows: Firstly, 1.25 mg of each copolymer obtained in this study was dissolved in 5 mL of THF and stirred for 12 h. Secondly, the deionized water was added dropwise to the solution under slight shaking, and then the solution was placed for 5 min to balance. After that, the light transmittance T% of the mixed solution was tested by UV–vis. When the T% was stable, the mixed solution was transferred to a dialysis membrane (MWCO 3500Da) to remove residual THF. The solution was dialyzed in deionized water with corresponding different pH values for 72 h. The particle size and morphology of the micelles were determined by DLS and SEM.

### 2.5. Preparation of DOX-Loaded Micelles

**Scheme 2:**
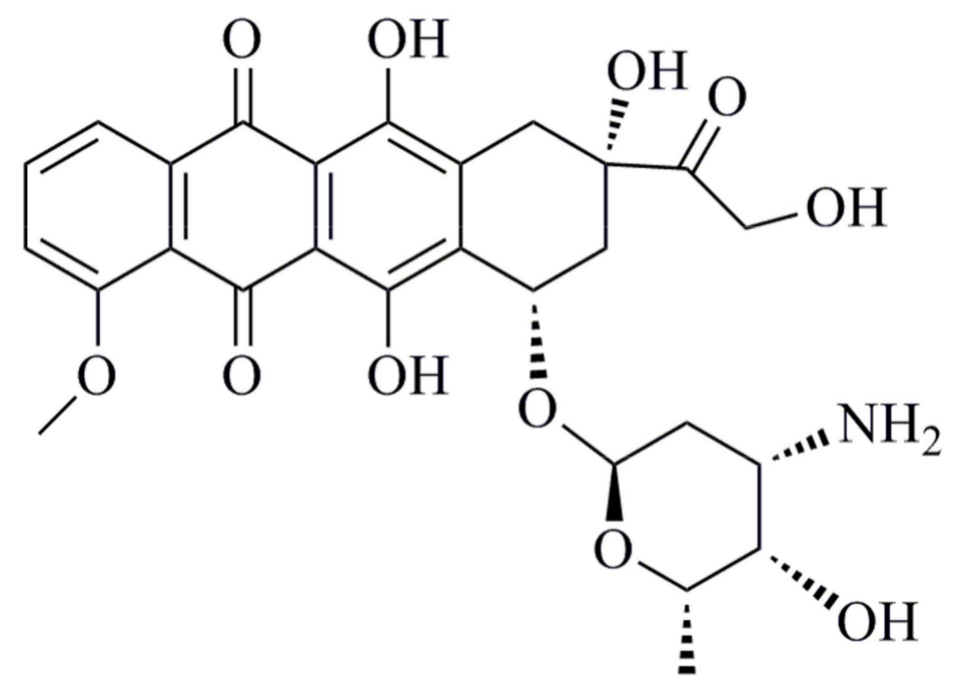
The chemical structure of DOX.

100 mg of the LC copolymers were dissolved in 20 mL of THF and put in a 50-mL flask. Then, 10 mg of DOX·HCl and 10 uL TEA were added. The mixed solution was stirred for 30 min at room temperature. After that, 20 mL of phosphate buffer was added dropwise and stirred for 2 h. Afterward, the excess drug and residual THF were removed by dialysis (MWCO 3500Da) for 24 h to obtain a DOX-loaded microsphere aqueous solution. The chemical structure of DOX is shown in Scheme 2. Next, the drug loading (DL%) and the entrapment efficiency (EE%) were calculated according to the following formulas [37]:

DL% = [(*C*_T_ − *C*_U_)/*C*_L_] × 100% (1)

EE% = (1 − *C*_U_/*C*_T_) × 100% (2)

where *C*_U_ is the weight of free unloaded drug, *C*_T_ is the total weight of drug added to the system, and *C*_L_ is the total weight of copolymer micelles. The specific process was as follows: part of the DOX-loaded microsphere aqueous solution was taken to freeze-dry at −80 °C, and lyophilized DOX-loaded micelles were dissolved in THF/DMSO (1:1, *v*/*v*), in which the structure of the micelles would be destroyed and the drug would be released completely. The transmittance (T%) of the mixed solution was monitored with UV–vis, and the drug content (*C*_T_−*C*_U_) could be calculated based on the standard curve and the proportion of samples taken. In addition, *C*_T_ and *C*_L_ were the weights of DOX and the LC copolymer added to prepare DOX-loaded micelles, respectively.

### 2.6. In Vitro Drug Release

DOX-release study was carried out in thermostat oscillator (37 °C, 80 rpm/min) in phosphate buffers of different pH, respectively: (a) pH = 6.4; (b) pH = 7.4; (c) pH = 8.4. In brief, 10 mL of DOX-loaded micelle aqueous solution was placed in a dialysis membrane (MWCO 3500Da). Then, the dialysis membrane was immersed into the abovementioned media and shaken at 37 °C. 5 mL of buffer sample was taken out periodically and 5 mL of fresh buffer was added, and the in vitro DOX release was analyzed using a UV–vis spectrometer at the absorbance of 481 nm.

## 3. Results and Discussion

### 3.1. Thermal Stability

**Figure 1:**
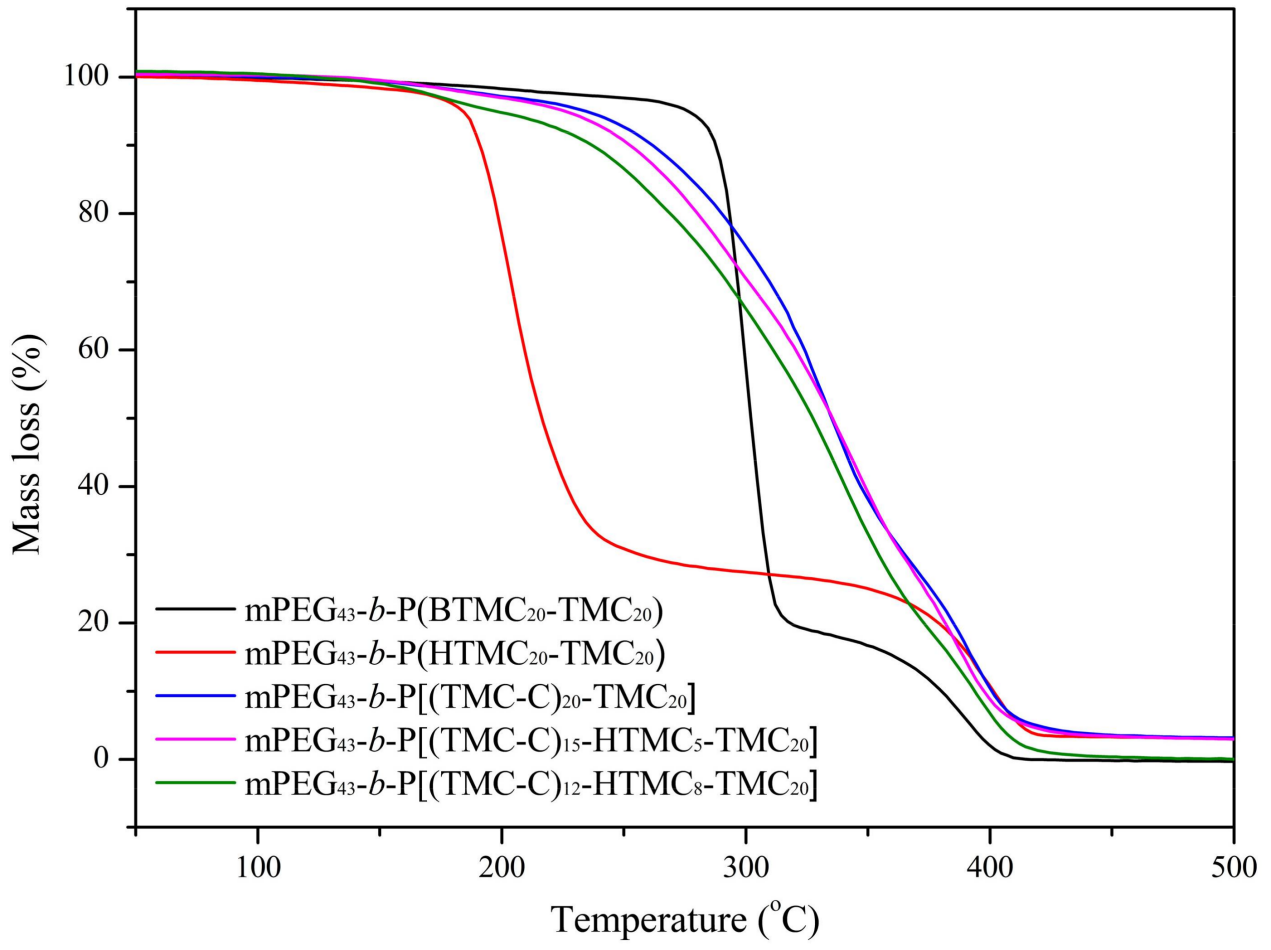
TGA curves of the copolymers.

The ^1^H NMR spectra of the three copolymers are showed in Figure S1. Figure 1 shows TGA curves of the block copolymers. The corresponding data of thermal decomposition and weight loss are summarized in Table 2.

In general, the decomposition temperature (*T*_d_) is the temperature at which 5% weight loss of copolymers occurred. According to Table 2, compared with mPEG_43_-*b*-P(BTMC_20_-TMC_20_), the *T*_d_ of *m*PEG_43_-*b*-P(HTMC_20_-TMC_20_) decreased by 92.3 °C. The main reason for this was that the existence of benzene groups enhanced intermolecular π–π conjugation so that the thermal stability of the copolymer increased. At the same time, the hydroxyl groups were more active and easily resulted in the occurrence of thermal decomposition. For the LC copolymers, when bulky cholesteryl units were introduced into side chains of the polycarbonate, the corresponding *T*_d_ increased. Furthermore, with a higher content of LC units, the LC copolymer was more stable. For example, *m*PEG_43_-*b*-P[(TMC-C)_20_-TMC_20_] containing the highest content of LC units revealed the highest *T*_d_ and the best stability. It suggested that the existence of LC units may cause a strong interaction between the repeating units and the polymer chains, which would enhance the stability of the copolymers.

### 3.2. Liquid Crystal Behavior

**Figure 2:**
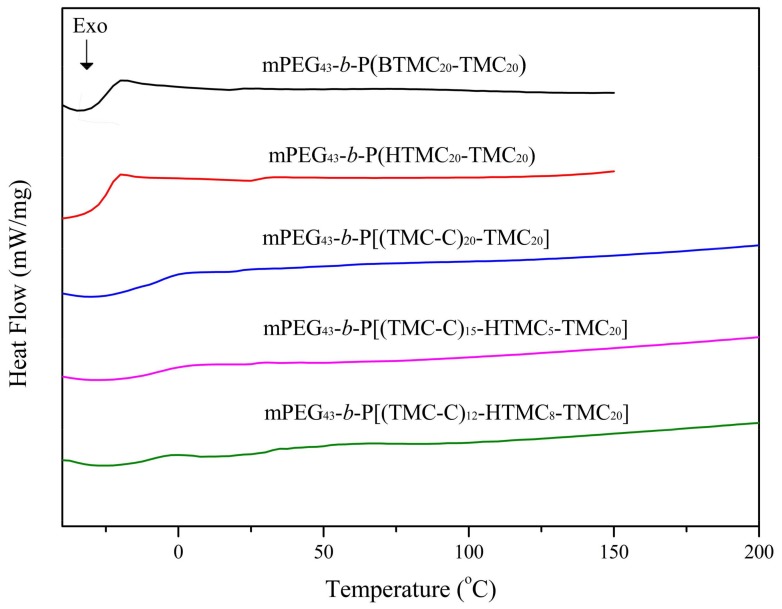
DSC curves of the copolymers.

The LC behavior of the copolymers was investigated with DSC, POM, and XRD. The corresponding glass transition temperatures (*T*_g_) obtained during the second heating cycles from DSC and the isotropic temperature (*T*_i_) obtained from POM are summarized in Table 3. Typical DSC curves of five copolymers are shown in Figure 2.

In general, the thermal properties of side-chain polymers mainly depend on the polymer backbone and the nature of the side groups. For all the copolymers with the same polycarbonate backbone in this investigation, the side groups had a significant influence on the glass transition temperature of the copolymers. As shown in Table 3, the corresponding *T*_g_ increased when strong-polarity hydroxyl groups were introduced into the polycarbonate backbone. In addition, when cholesteryl units were introduced, the corresponding *T*_g_ also increased because the copolymers had a higher average molecular weight and more rigid side-chain cholesteryl groups. As seen in Figure 2, the copolymer *m*PEG_43_-*b*-P(HTMC_20_-TMC_20_) with side hydroxyl groups showed *T*_g_ at −24.1 °C, while the three LC copolymers with side-chain cholesteryl groups *m*PEG_43_-*b*-P[(TMC-C)_12_-HTMC_8_-TMC_20_], *m*PEG_43_-*b*-P[(TMC-C)_15_-HTMC_5_-TMC_20_], and *m*PEG_43_-*b*-P[(TMC-C)_20_-TMC_20_] showed *T*_g_ at −11.2 °C, −7.4 °C, and −4.6 °C, respectively. It suggested that the copolymers containing LC units exhibited LC phases at human body temperature. Therefore, they may be used clinically as self-assembling materials with orientational order.

**Figure 3:**
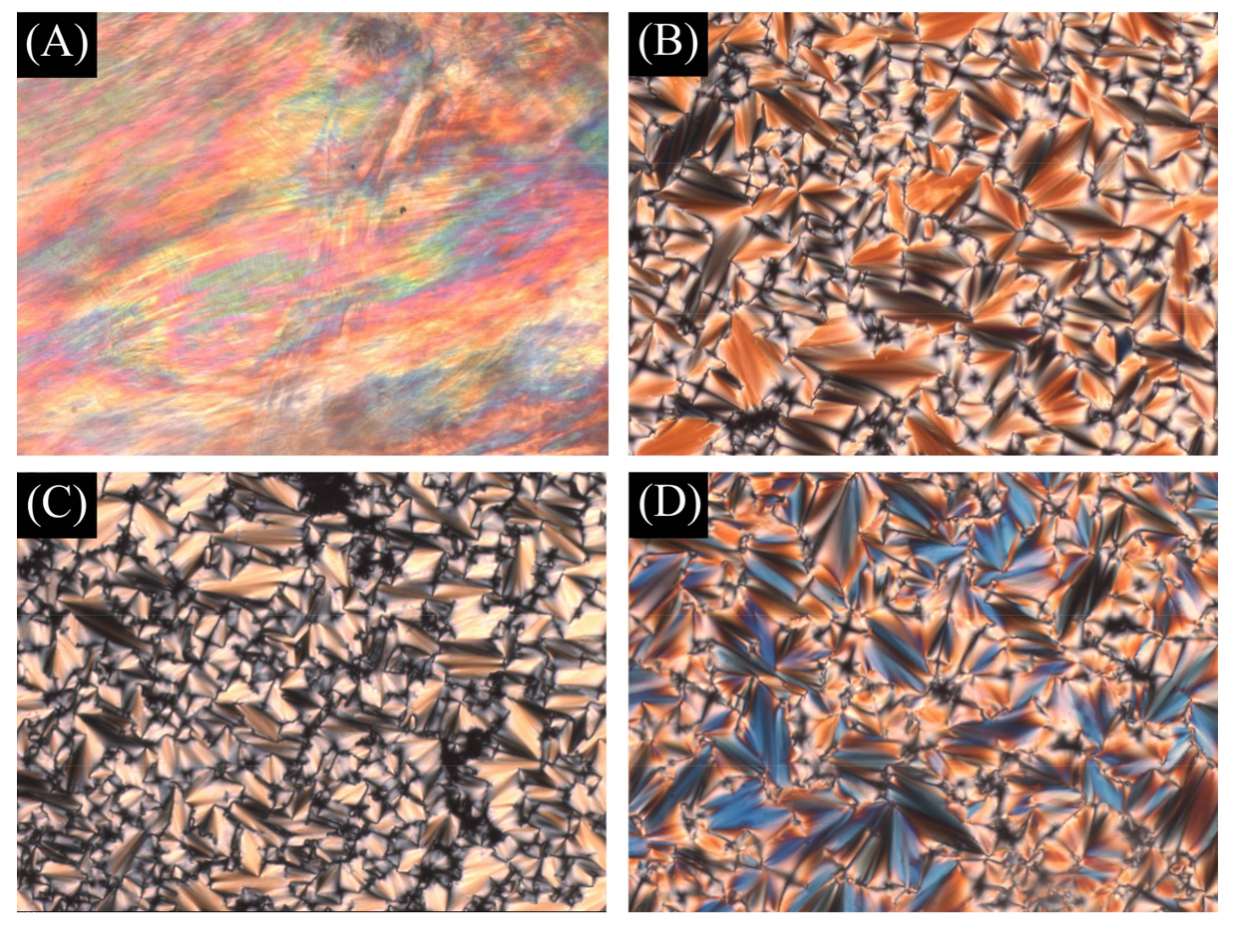
Polarizing optical microscope (POM) texture of the LC copolymers (200×). Polarizing optical microscope (POM) texture of the LC copolymers. (**A**) LC texture of a SmA phase at 40 °C for mPEG_43_-*b*-P[(TMC-C)_20_-TMC_20_]; (**B**) fan-shaped texture of a SmA phase at 116 °C for mPEG_43_-*b*-P[(TMC-C)_20_-TMC_20_]; (**C**) fan-shaped texture of a SmA phase at 120 °C for mPEG_43_-*b*-P[(TMC-C)_15_-HTMC_5_-TMC_20_]; (**D**) fan-shaped texture of a SmA phase at 104 °C for mPEG_43_-*b*-P[(TMC-C)_12_-HTMC_8_-TMC_20_].

For the LC polymers, the formation of the mesophase may be influenced by the polymer backbone, aspect ratio of the mesogen, flexible spacer, and polarity of the molecules [40,41]. Optical textures of the three LC copolymers are shown in Figure 3. POM showed that all the LC copolymers exhibited the fan-shaped texture of a smectic A (SmA) phase in heating and cooling processes. According to the results reported by Hu et al. [29], the corresponding chiral monomer C showed a cholesteric phase. The result indicated that the macromolecular chain might hinder the formation of a cholesteric helical supermolecular structure and an ordered organization into the mesophase.

**Figure 4:**
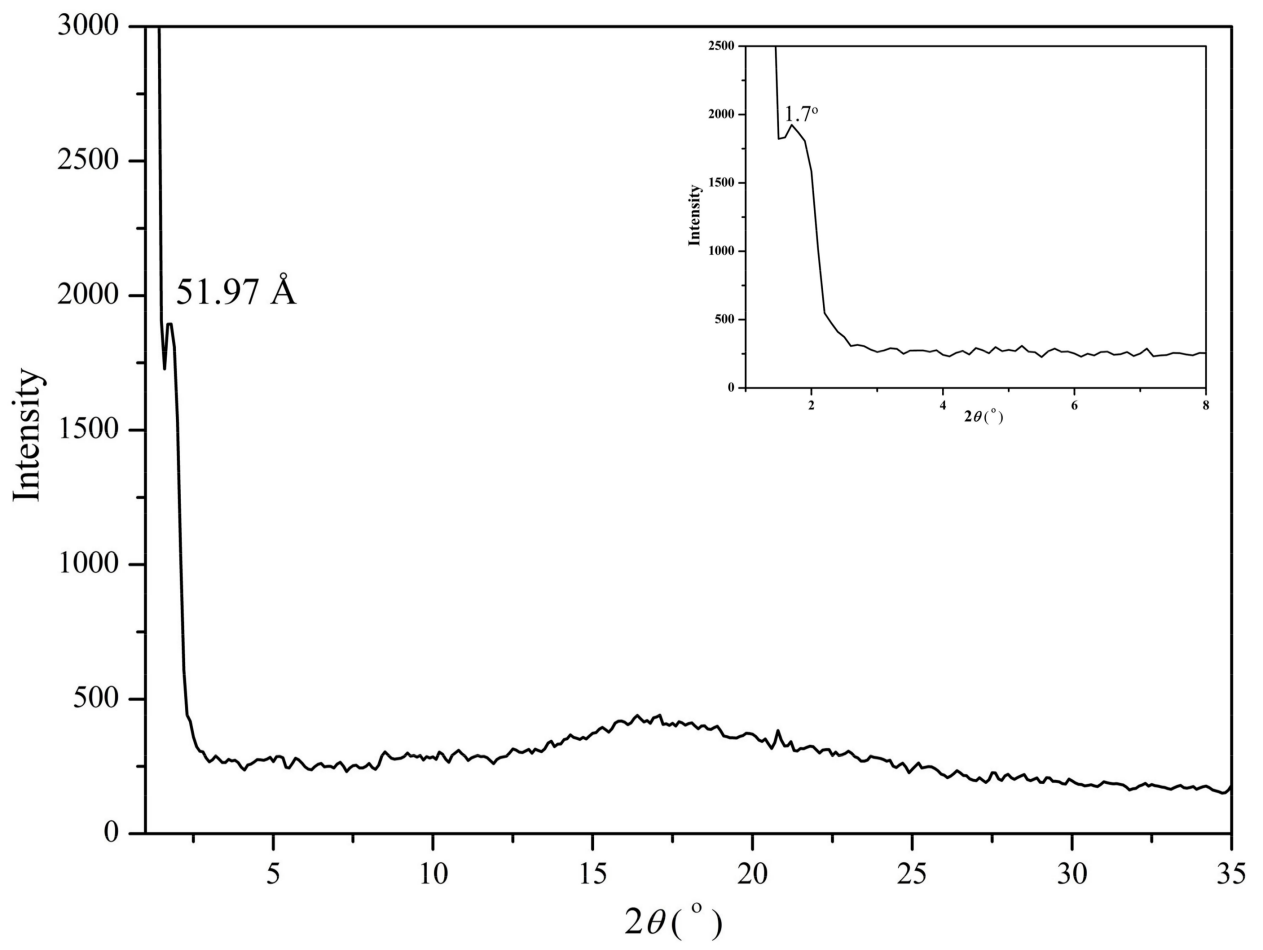
X-ray diffraction (XRD) pattern of mPEG_43_-*b*-P[(TMC-C)_20_-TMC_20_] at 120 °C.

**Figure 5:**
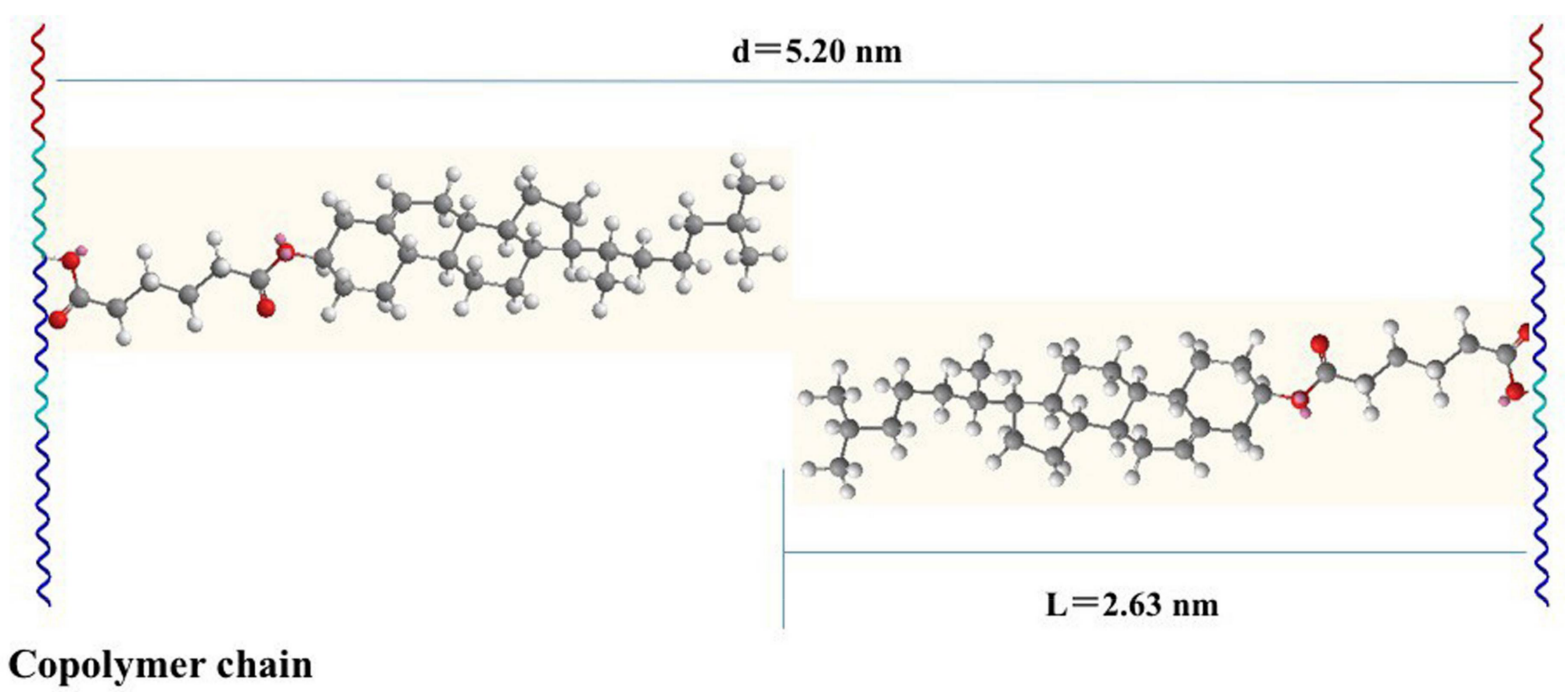
Schematic representation of the doubled SmA layer arrangements of mPEG_43_-*b*-P[(TMC-C)_20_-TMC_20_].

XRD is a powerful instrument for the identification of mesophase structure. XRD measurements of the LC copolymers were carried out at the mesophase temperature of 120 °C. As an example, the XRD pattern of mPEG_43_-*b*-P[(TMC-C)_20_-TMC_20_] is shown in Figure 4. The XRD pattern showed a small-angle reflection and a broad diffuse peak at a wide angle. In general, a sharp reflection in the small-angle region, corresponding to the periodic distance, is characteristic of a smectic phase. Combined with POM texture, the LC copolymers obtained in this study can be judged as having a SmA phase. According to Figure 4, the molecular layer spacing *d* was calculated to be 51.97 Å. The all-trans molecular length *L* of the most extended conformation of mPEG_43_-*b*-P[(TMC-C)_20_-TMC_20_] was about 26.3 Å, which could be calculated by using ChemBio3D-Ultra and MM2 minimal energy parameters. A *d*/*L* ratio of 1.97 (d ≈ 2*L*) was calculated, indicating an orderly doubled arrangement of mPEG_43_-*b*-P[(TMC-C)_20_-TMC_20_]. This partial bilayer structure was similar to that of a SmA_d_ phase formed by polar mesogens. Similar results also have been reported [42,43]. The possible molecular arrangement model of the doubled SmA layer for mPEG_43_-*b*-P[(TMC-C)_20_-TMC_20_] is shown in Figure 5.

### 3.3. Self-Assembly Behavior

**Figure 6:**
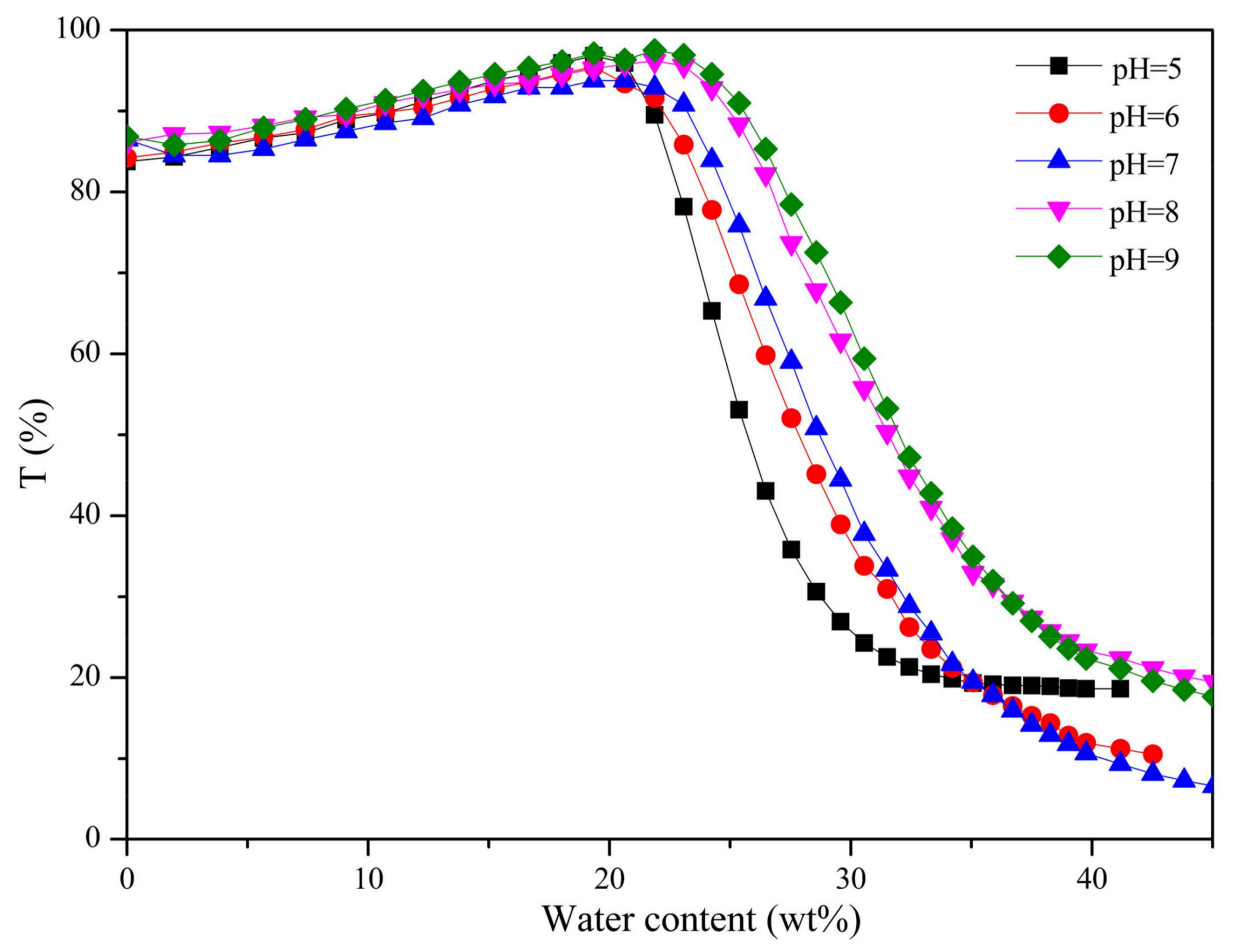
Relationships between light transmission at 267 nm and water content of mPEG_43_-*b*-P[(TMC-C)_20_-TMC_20_] for the self-assembly process at different pH levels.

To research the self-assembly behavior of the LC copolymers, the classic nanoprecipitated self-assembly or dialysis method is used with different acidic environments. The block copolymer mPEG_43_-*b*-P[(TMC-C)_20_-TMC_20_] was first dissolved in THF in five vials equipped with a sealing cap, and then aqueous solutions with different pH values were added dropwise, respectively. The light transmittance of mixture solutions with different water content was determined by UV–vis at 267 nm, and the corresponding relationships are shown in Figure 6.

When the water content was less than 20 wt %, the mixed solution remained transparent and the light transmittance changed a little. With the increase of water content, the light transmittance of the mixed solution revealed a slowly reduced trend, suggesting that the copolymer began to form micelles. The light transmittance was stable when the water content reached 40 wt %, indicating that the formation of micelles was nearly finished. Figure 6 shows that the self-assembly process speeded up gradually with the decrease of the pH value. There were hydrogen bonding forces present at pH = 7.0, and the hydrophilia of kernels was enhanced due to the protonation of hydroxyl groups in a weak-acid condition, and both of these would accelerate the self-assembly process.

After the formation of stable aggregates, the mixture solutions were dialyzed for 72 h under room temperature to remove THF completely, and then the particle size and distribution of the self-assembled micelles were characterized by DLS; the results are shown in Table 4. Particle dispersion of the self-assembled micelles was about 0.1–0.2 at pH 5–9, indicating that the size distribution was relatively uniform. When pH > 7, the size of particle changed a little because the deprotonation of the –OH groups in copolymer would occur and result in the disruption of hydrogen bonds and the enhancement of the hydrophobicity of the copolymer. As the pH value decreased, the particle size increased. These results showed that the hydrophilia of kernels was enhanced because of the protonation of hydroxyl groups. Moreover, the mutual exclusion of the proton segment with stronger positive charge may promote the micellar swelling and further result in the increase of particle size.

**Figure 7:**
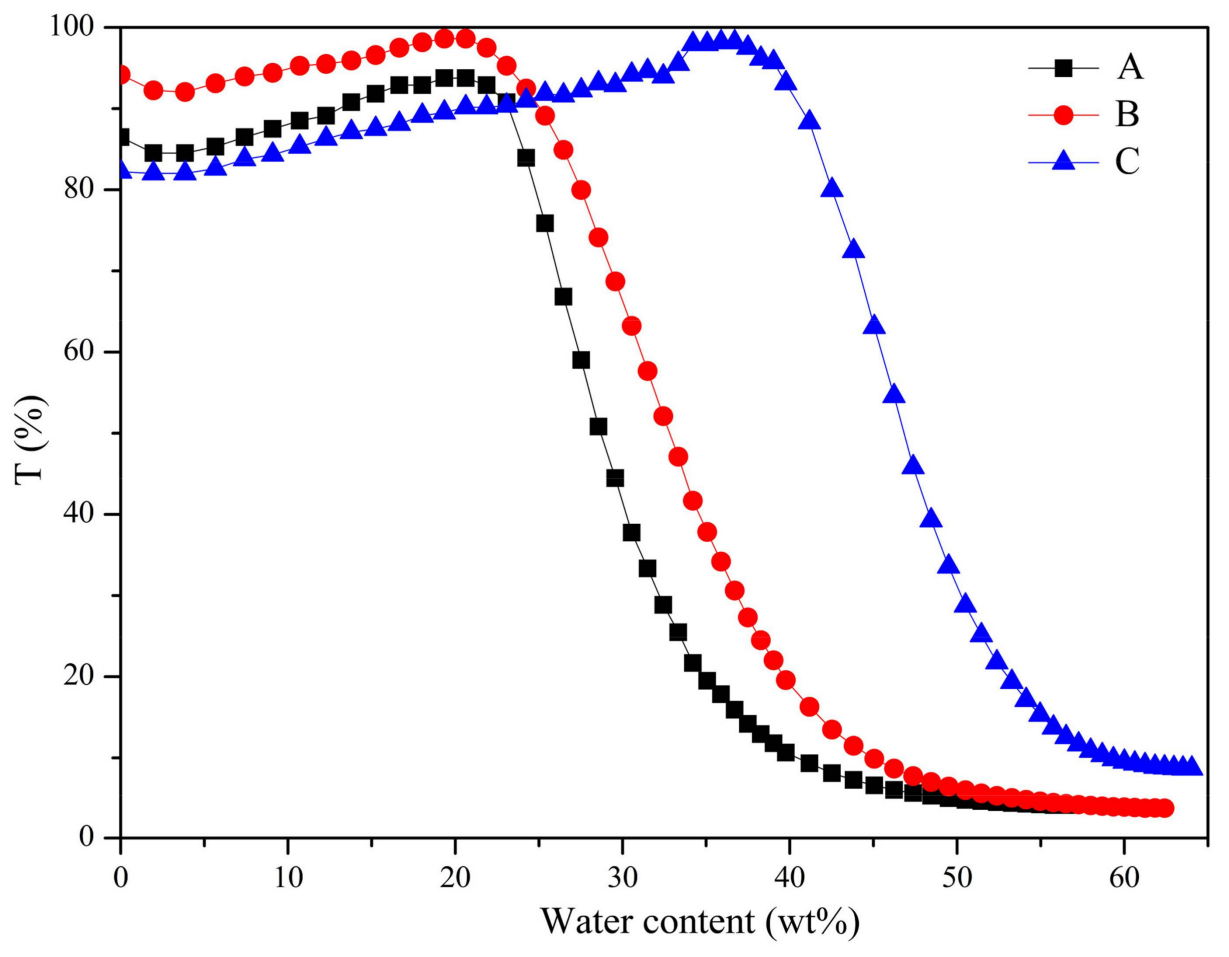
Relationships between light transmission at 267 nm and water content for the self-assembly behavior of the different copolymer structures (A) *m*PEG_43_-*b*-P[(TMC-C)_20_ TMC_20_]; (B) *m*PEG_43_-*b*-P[(TMC-C)_15_-HTMC_5_-TMC_20_]; and (C) mPEG_43_-*b*-P[(TMC-C)_12_-HTMC_8_-TMC_20_].

Three LC copolymers were dissolved in THF and assembled in the deionized water at pH = 7. The relationships between light transmittance and the water content of mixture solution were determined by UV–vis and shown in Figure 7. The LC copolymers *m*PEG_43_-*b*-P[(TMC-C)_20_-TMC_20_] and *m*PEG_43_-*b*-P[(TMC-C)_15_-HTMC_5_-TMC_20_] began to assemble when the water content was about 20 wt %, and became stable as the water content reached 41 wt % and 46 wt %, respectively. As for *m*PEG_43_-*b*-P[(TMC-C)_12_-HTMC_8_-TMC_20_], which contained fewer LC units, it began to assemble at a water content of 45 wt %, and remained stable until the water content reached 59 wt %. Therefore, the LC content played an important role in the self-assembly behavior. The higher the LC content, the faster the self-assembly process was. It was because of the stronger driving force for self-assembly caused by the improved orientation ability of copolymers as the LC content increased.

As shown in Table 4, the aggregate size of the LC block copolymers was smaller with increasing LC unit content. The introduction of LC units would lead to the decrease of hydroxyl groups in the copolymer, and the copolymers became more hydrophobic. In addition, as the LC unit content increased, the orientation of the molecule would be enhanced to provide a stronger driving force for self-assembly, and produce a more compact structure, resulting in smaller particle size.

**Figure 8:**
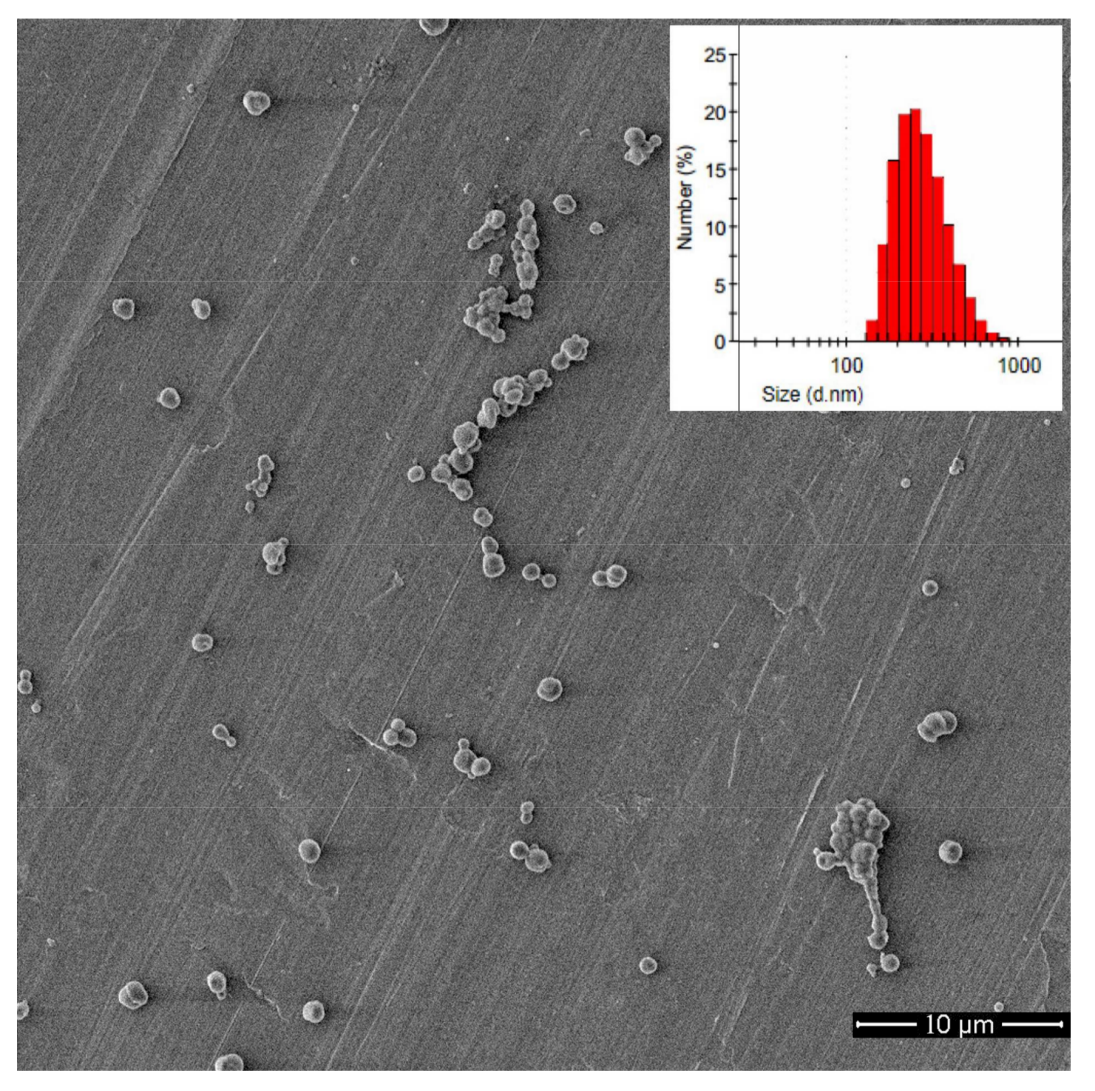
Scanning electron microscope (SEM) image of the self-assembled aggregates for *m*PEG_43_-*b*-P[(TMC-C)_20_ TMC_20_] in pH = 7.

The morphologies of the *m*PEG_43_-*b*-P[(TMC-C)_20_-TMC_20_] micelles were determined by SEM, and presented in Figure 8. The results indicated that the aggregates exhibited spherical micelles. The LC copolymers were dissolved in THF solution and the chain was stretched. When the selective solvent of water was added, the polycarbonates with LC side segments quickly froze due to the hydrophobicity, and THF was pumped continuously; as a result the micelles formed gradually. However, in this process, the micelle structure was likely to continue adjusting because the molecules in the LC state possessed orientation ability with the addition of water. Thus, the copolymer made further arrangements neatly and the inner space of the hydrophobic kernel was more compact, and a solid sphere aggregate formed, finally. Since there was a certain liquidity of LCs, microsphere fusion appeared, as shown in Figure 8.

### 3.4. DOX-Loaded Micelles

**Figure 9:**
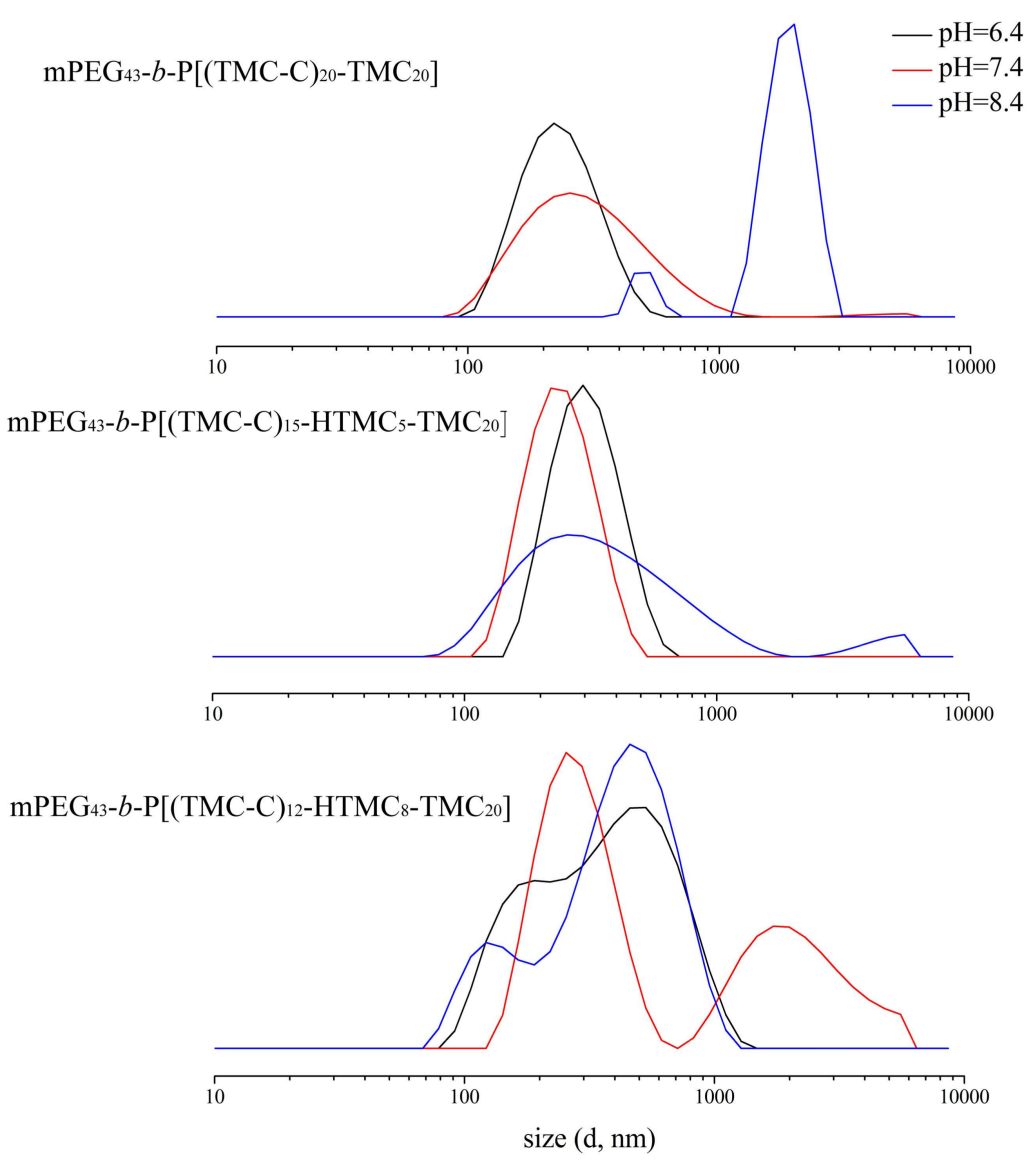
pH-responsive particle sizes of the DOX-loaded copolymer micelles at 37 °C in phosphate buffer (pH = 6.4, 7.4, 8.4) by DLS.

Figure 9 depicts particle size and distribution situation of DOX-loaded micelles characterized by DLS, and the detailed data are listed in Table 5.

The copolymer structure and the pH value had a critical influence on the DOX-loading efficiency and the particle size of the drug-loaded micelles. As shown in Figure 9 and Table 5, the particle size of DOX-loaded *m*PEG_43_-*b*-P[(TMC-C)_12_-HTMC_8_-TMC_20_] tended to form double peaks in the self-assembly process, and the size of DOX-loaded *m*PEG_43_-*b*-P[(TMC-C)_12_-HTMC_8_-TMC_20_] was larger than that of other copolymers. It was ascribed to the lowest content of hydrophobic LC units of *m*PEG_43_-*b*-P[(TMC-C)_12_-HTMC_8_-TMC_20_] making it the most hydrophilic, resulting in the swelling of the corresponding microspheres. Furthermore, the size of DOX-loaded *m*PEG_43_-*b*-P[(TMC-C)_12_-HTMC_8_-TMC_20_] micelles was larger at pH = 7.4 than that at pH = 6.4 and pH = 8.4 because of the maximum drug loading. However, the size of DOX-loaded *m*PEG_43_-*b*-P[(TMC-C)_15_-HTMC_5_-TMC_20_] micelles was minimum at pH = 7.4. Here are some reasons: Firstly, the strong hydrogen bond force between the copolymers and DOX-loaded microspheres caused them to shrink closely together. Secondly, the copolymer *m*PEG_43_-*b*-P[(TMC-C)_15_-HTMC_5_-TMC_20_], with more LC units and its hydrophobic core, arranged neatly and packed tightly so as to form a smaller particle size of microspheres. However, when the content of LC units in the copolymers continued to increase, the particle size also increased, owing to the increase of molecular volume. This is why the particle size of DOX-loaded *m*PEG_43_-*b*-P[(TMC-C)_20_-TMC_20_] (256.6 nm) at pH = 7.4 was larger than that of DOX-loaded *m*PEG_43_-*b*-P[(TMC-C)_15_-HTMC_5_-TMC_20_] (225.2 nm).

Compared to other copolymers, mPEG_43_-*b*-P[(TMC-C)_12_-HTMC_8_-TMC_20_] showed the lowest DOX-loading efficiency under the conditions of pH ≤ 7.4. As the most hydrophilic copolymer, it was difficult to load more hydrophobic drug, such as DOX.

The DOX loading of all copolymer micelles reached the maximum value when pH = 7.4, owing to the hydrogen bond force between copolymers and DOX being strongest at pH = 7.4. Furthermore, the drug loading of the three copolymers all exhibited a decreasing trend in an alkaline condition. The results showed that DOX had a low solubility under an alkaline condition, and the copolymer was ionized which gave it a certain repulsion towards DOX, causing less DOX to be loaded into the micelle core finally.

**Figure 10:**
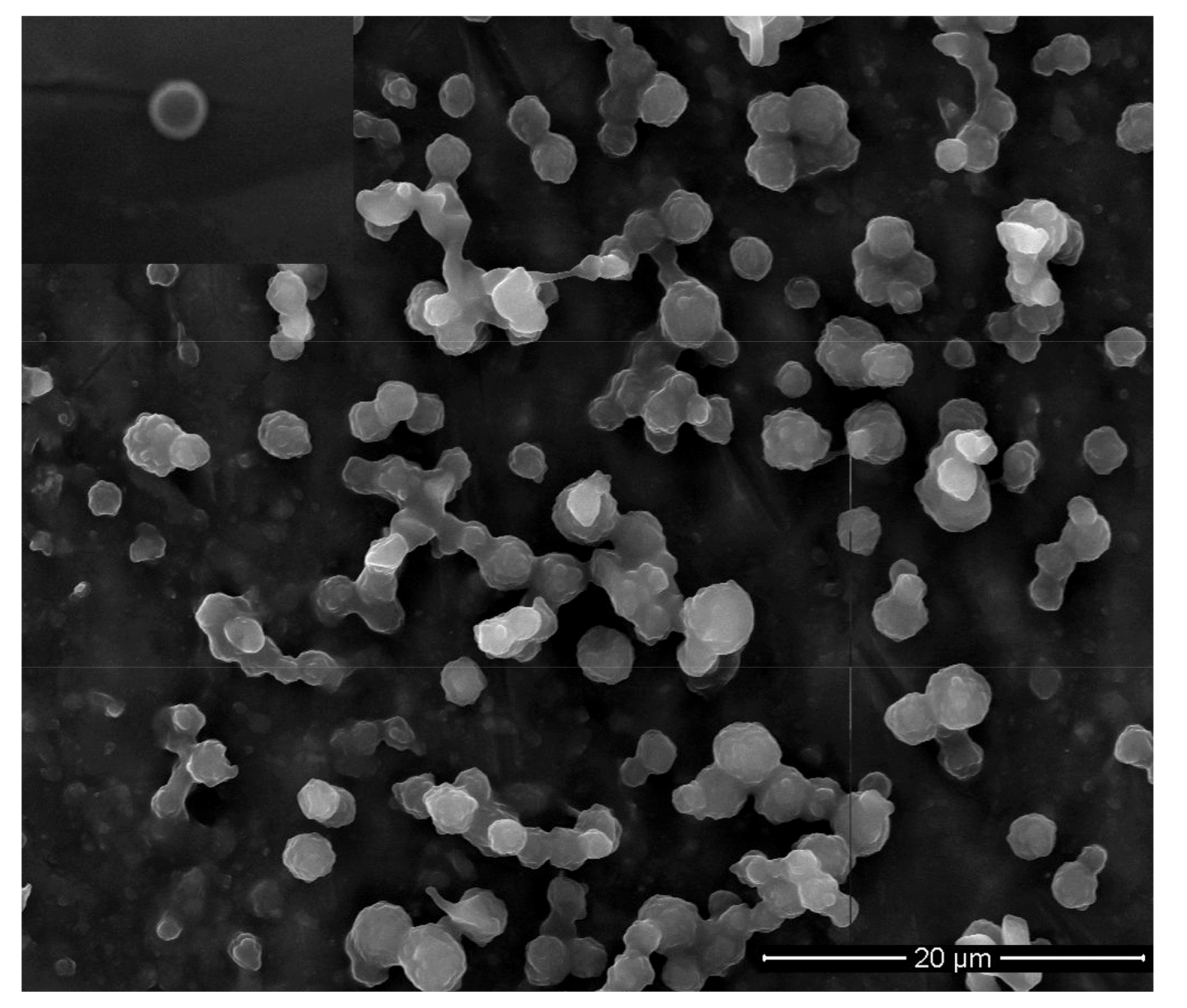
SEM image of DOX-loaded mPEG_43_-*b*-P[(TMC-C)_20_-TMC_20_] micelles.

The morphologies of DOX-loaded copolymer micelles were characterized by SEM. The morphology of *m*PEG_43_-*b*-P[(TMC-C)_20_-TMC_20_] micelles is typical, as shown in Figure 10. The drug-loaded copolymers showed spherical and irregular shapes, which was similar to micelles without DOX. However, it was surprising that the size of the micelles showed a decreased trend after loading of the drug. The reason may be that strong hydrogen bond forces between the copolymers and DOX shrink the particle size.

### 3.5. In Vitro DOX Release from the Micelles

**Figure 11:**
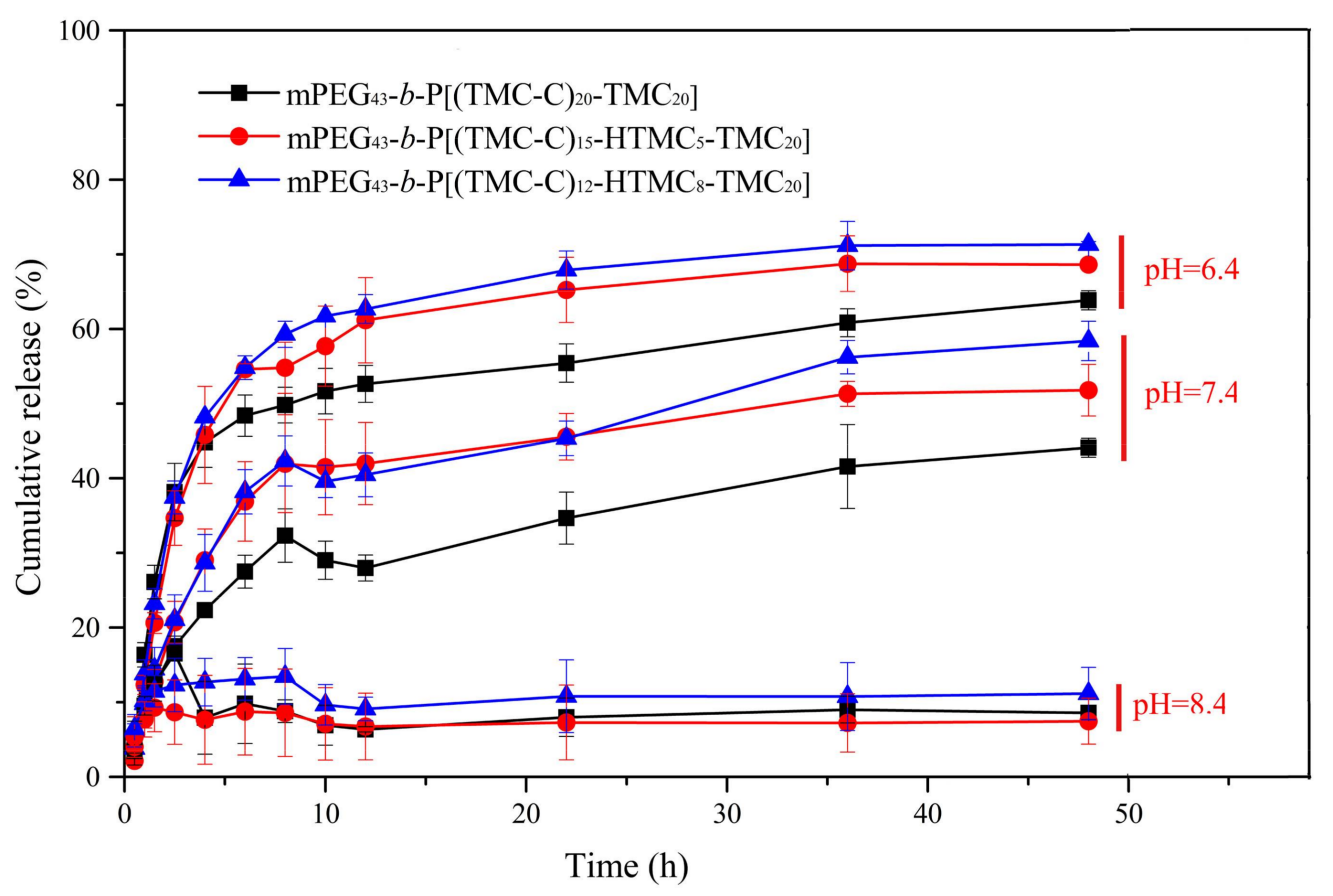
DOX release profiles of the LC copolymer micelles at 37 °C in PBS at different pH levels.

In vitro DOX release profiles of the copolymers *m*PEG_43_-*b*-P[(TMC-C)_20_-TMC_20_], *m*PEG_43_-*b*-P[(TMC-C)_15_-HTMC_5_-TMC_20_], and *m*PEG_43_-*b*-P[(TMC-C)_12_-HTMC_8_-TMC_20_] in various media are shown in Figure 11. And the simulation diagram of DOX loading and realease behavior is showed in Figure S2.

At pH = 8.4, the drug within the three drug-loaded copolymer micelles was slowly released, and only 10% DOX was released in the end. At pH = 7.4, the DOX release rate was obviously accelerated, and its cumulative release could reach 40–60% within 48 h. The cumulative DOX release could increase up to 60–70% when the pH was decreased to 6.4. For *m*PEG_43_-*b*-P[(TMC-C)_20_-TMC_20_], 63%, 44%, and 8% DOX was released within 48 h at pH = 6.4, 7.4, and 8.4, respectively. The results could be explained as due to the fact that the molecular protonation extent was stronger under acidic conditions, and the water-solubility of DOX and copolymers increased as well, so ultimately, the DOX release rate was accelerated.

The cumulative DOX release at pH = 7.4 for the drug micelles of *m*PEG_43_-*b*-P[(TMC-C)_15_-HTMC_5_-TMC_20_] and mPEG_43_-*b*-P[(TMC-C)_12_-HTMC_8_-TMC_20_] was 51% and 58%, respectively. The experimental results showed that the existence of LC units could reduce the release rate of DOX to some extent.

## 4. Conclusions

In summary, three new LC amphipathic polycarbonate copolymers containing side-chain cholesteryl units were synthesized and characterized. The LC copolymers all showed high thermal stability, exhibited a fan-shape texture, and revealed a double mesogenic molecular arrangement of a SmA phase. In addition, with the increase of LC unit content in the copolymers, the corresponding *T*_m_, *T*_i_, and *T*_d_ increased. In the process of self-assembly, the aggregates of the LC copolymers exhibited spherical micelles with pH responsiveness. Moreover, the copolymers containing higher contents of LC units tended to form smaller microspheres. The LC copolymers were taken as carriers in which to load DOX, and study the drug loading capacity of the copolymers and in vitro drug release of micelles. The drug loading capacity was best at pH = 7.4. The size of the drug-loaded copolymer microspheres was not only related to the pH value, but also related to the contents of LC units in the copolymers. The drug release process also had a pH response, and DOX could be released quickly in a weakly acidic environment, which is expected to enable it to be used clinically for applications in drug delivery to specific tumor sites.

## Figures and Tables

**Table 1:** Fabrication of mPEG_43_-*b*-P[(TMC-C)_20−*x*_-HTMC*_x_*-TMC*_y_*] and 6-cholesteroxy-6-oxocaproic acid.

Copolymer	*m* (–OH)	*n* (–OH)	*m* (–COOH)	*n* (–COOH)
	g	mol	g	mol
*m*PEG_43_-*b*-P[(TMC-C)_20_-TMC_20_]	5.00	0.016	8.28	0.016
*m*PEG_43_-*b*-P[(TMC-C)_15_-HTMC_5_-TMC_20_]	5.00	0.016	6.21	0.012
*m*PEG_43_-*b*-P[(TMC-C)_12_-HTMC_8_-TMC_20_]	5.00	0.016	3.58	0.008

**Table 2:** TGA data of the copolymers.

Copolymer	*T*_d_^a^ (°C)	Weight Loss (%)				
		200 °C	250 °C	300 °C	350 °C	400 °C
*m*PEG_43_-*b*-P(BTMC_20_-TMC_20_)	276.5	2.04	3.40	42.83	83.68	98.35
*m*PEG_43_-*b*-P(HTMC_20_-TMC_20_)	184.2	23.19	69.22	72.63	75.04	89.32
*m*PEG_43_-*b*-P[(TMC-C)_20_-TMC_20_]	234.4	3.31	7.79	25.31	62.36	90.09
*m*PEG_43_-*b*-P[(TMC-C)_15_-HTMC_5_-TMC_20_]	225.8	3.45	9.74	29.86	61.31	91.62
*m*PEG_43_-*b*-P[(TMC-C)_12_-HTMC_8_-TMC_20_]	197.2	6.15	14.33	34.86	67.91	94.21

**Table 3:** Thermal properties of the copolymers.

Copolymer	Phase Transition Temperature (°C)
*m*PEG_43_-*b*-P(BTMC_20_-TMC_20_)	g −25.0 I
*m*PEG_43_-*b*-P(HTMC_20_-TMC_20_)	g −24.1 I
*m*PEG_43_-*b*-P[(TMC-C)_20_-TMC_20_]	g −4.6 SmA 180.0 I
*m*PEG_43_-*b*-P[(TMC-C)_15_-HTMC_5_-TMC_20_]	g −7.4 SmA 169.8 I
*m*PEG_43_-*b*-P[(TMC-C)_12_-HTMC_8_-TMC_20_]	g −11.2 SmA 142.6 I

g: glass state; SmA: smectic A phase; I: isotropic.

**Table 4:** Self-assembled particle size distribution and PDI of micelles at different pH levels.

Copolymer	pH	Z-Average, d/nm	PDI
**P1**	5	346.3	0.136
**P1**	6	307.4	0.123
**P1**	7	296.4	0.146
**P1**	8	295.1	0.149
**P1**	9	301.7	0.179
**P2**	7	313.4	0.132
**P3**	7	352.9	0.067

P1: mPEG_43_-*b*-P[(TMC-C)_20_-TMC_20_]; P2: mPEG_43_-*b*-P[(TMC-C)_15_-HTMC_5_-TMC_20_]; P3: mPEG_43_-*b*-P[(TMC-C)_12_-HTMC_8_-TMC_20_]; PDI: the distribution of the particle size.

**Table 5:** Particle size and the DOX-loading efficiency for the drug-loaded copolymer micelles.

Copolymer	P1			P2			P3		
	pH = 6.4	pH = 7.4	pH = 8.4	pH = 6.4	pH = 7.4	pH = 8.4	pH = 6.4	pH = 7.4	pH = 8.4
Size (nm)	219.0	256.6	304.5	286.3	225.2	298.2	319.6	398.6	305.6
PDI	0.112	0.224	0.506	0.083	0.071	0.312	0.322	0.428	0.253
DL (%)	0.544	0.541	0.226	0.484	0.572	0.343	0.447	0.508	0.360
EE (%)	33.21	31.82	13.20	32.92	32.62	20.74	28.92	27.62	21.72

P1: mPEG_43_-*b*-P[(TMC-C)_20_-TMC_20_]; P2: mPEG_43_-*b*-P[(TMC-C)_15_-HTMC_5_-TMC_20_]; P3: mPEG_43_-*b*-P[(TMC-C)_12_-HTMC_8_-TMC_20_]; DL: drug loading efficiency; EE: entrapment efficiency.

## Supplementary Materials

The following are available online at http://www.mdpi.com/2079-4991/8/4/195/s1. Synthesis and structural characterization: Figure S1: ^1^H NMR spectra of LC copolymers. A: mPEG_43_-*b*-P[(TMC-C)_20_-TMC_20_]; B: mPEG_43_-*b*-P[(TMC-C)_15_-HTMC_5_-TMC_20_]; C: mPEG_43_-*b*-P[(TMC-C)_12_-HTMC_8_-TMC_20_]; Figure S2: The simulation diagram of DOX loading and release behavior.
