# Supplementary material for: Synthesis, Self-Assembly, and Drug-Release Properties of New Amphipathic Liquid Crystal Polycarbonates
Source: Nanomaterials (Basel). 2018 Mar 27;8(4):195. doi: 10.3390/nano8040195 (PMC5923525; doi:10.3390/nano8040195)
Supplement: Supplementary file 1 [file nanomaterials-08-00195-s001.pdf]

Supporting Information for

Synthesis, self-assembly and drug release properties of new amphipathic liquid crystal polycarbonates

Yu-Jiao Xie [1], Xiao-Feng Liu [1], Zhuang Hu [1], Zhi-Peng Hou [1], Zhi-Hao Guo [1], Zhang-Pei Chen [1], Jian-She Hu [1,*] and Li-Qun Yang [2,*]

[1] Center for Molecular Science and Engineering, College of Science, Northeastern University, Shenyang, 110819, P. R.China; xieyujiao5819573@gmail.com (Y.-J.X.); 1510048@stu.neu.edu.cn (X.-F.L.); 1710059@stu.neu.edu.cn (Z.H); 1670175@stu.neu.edu.cn (Z.-P.H); 1610046@stu.neu.edu.cn (Z.-H.G.); chenzhangpei@mail.neu.edu.cn (Z.-P.C.)

[2] Key Laboratory of Reproductive Health, Liaoning Research Institute of Family Planning, Shenyang, 110031, P.R.China.

[*] Correspondence: hujs@mail.neu.edu.cn; yanglq@lnszjk.com.cn.

Synthesis and structural characterization

It is well known that, the ring-opening polymerization is an efficient method to prepare biodegradable aliphatic polycarbonate. In this study, five new block copolymers were synthesized according to the route shown in Scheme 1. First, amphiphilic block copolymer mPEG$_{43}$-*b*-P(BTMC$_{20}$-TMC$_{20}$) was synthesized by the copolymerization of BTMC and TMC using mPEG as initiator and Sn(Oct)$_2$ as catalyst. Secondly, the protective benzyl groups in polymer side chains were removed using Pd/C and Pd(OH)$_2$/C as co-reductant under hydrogen atmosphere to obtain mPEG$_{43}$-*b*-P(HTMC$_{20}$-TMC$_{20}$). Finally, the chrial monomer 6-cholesteroxy-6-oxocaproic acid was reacted with mPEG$_{43}$-*b*-P(HTMC$_{20}$-TMC$_{20}$) using DCC as condensation agent and DMAP as catalyst to obtain three new amphiphilic LC copolymers mPEG$_{43}$-*b*-P[(TMC-C)$_{20}$-TMC$_{20}$], mPEG$_{43}$-*b*-P[(TMC-C)$_{15}$-HTMC$_5$-TMC$_{20}$] and mPEG$_{43}$-*b*-P[(TMC-C)$_{12}$-HTMC$_8$-TMC$_{20}$]. The chemical structures of five block copolymers obtained were confirmed by FT-IR and ^{1}H NMR spectra. After ring-opening polymerization, the corresponding signals of methylene proton at 4.47 ppm for BTMC and at 2.13 ppm for TMC shifted to 4.25 ppm and 2.03 ppm in mPEG$_{43}$-*b*-P(BTMC$_{20}$-TMC$_{20}$). In addition, the peak area of 2.03 ppm was compared with that of 4.25 ppm, it could be found that the content of TMC and BTMC was equal. And ^{1}H NMR spectra of mPEG$_{43}$-*b*-P(HTMC$_{20}$-TMC$_{20}$) indicated that the proton signals of benzyl groups at 7.26 ppm and 4.65 ppm disappeared as well as new proton signal of hydroxyl groups at 5.45 ppm appeared. Figure S1 shows ^{1}H NMR spectra of mPEG$_{43}$-*b*-P[(TMC-C)$_{20-x}$-HTMC$_x$-TMC$_y$]. The corresponding proton signals of mPEG and polycarbonate chains, and side cholesteryl units could be observed. In addition, when the molar ratio of the polycarbonate and LC monomer was 1:1, the peak area ratio of proton at 5.37 ppm for –C***H***=C in cholesteryl and 5.27 ppm for OCH$_2$–C***H***–CH$_2$O– in PHTMC was 1:0.97. As the molar ratio increased, the peak area ratio increased to 1:0.77 and 1:0.61, respectively. The number

average molecular weight (M_n) and polydispersity index (PDI) were detected by GPC. Average molecular weight of mPEG$_{43}$-*b*-P(BTMC$_{20}$-TMC$_{20}$) was 8013 and PDI was 1.57. Polymerization degree of mPEG$_{43}$-*b*-P(BTMC$_{20}$-TMC$_{20}$) was calculated by combining the results of GPC with ^{1}H NMR.

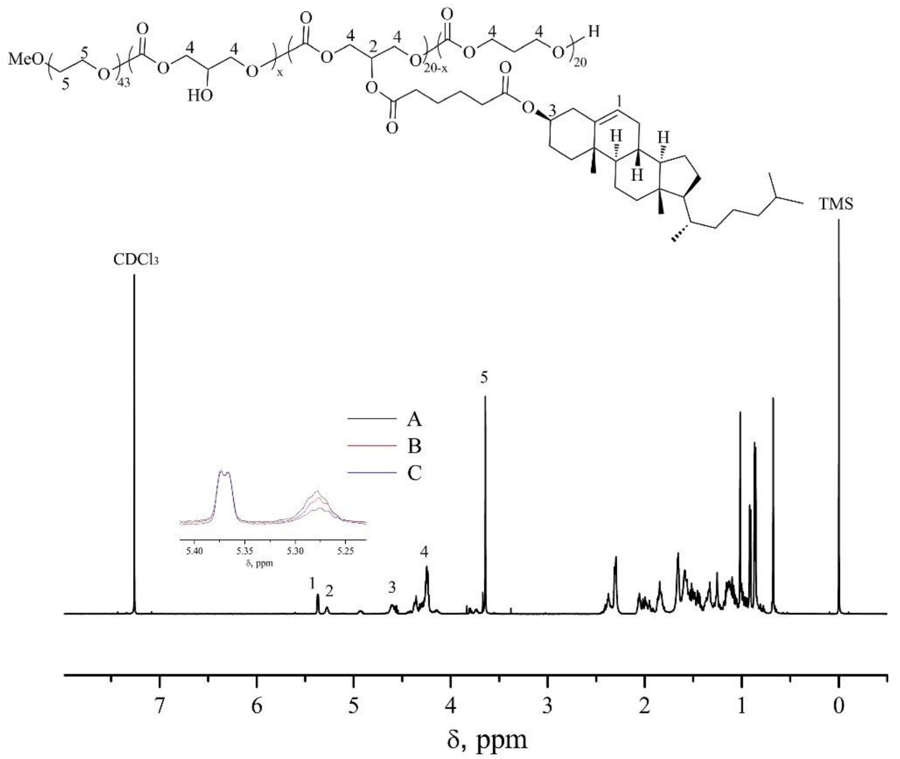

Figure S1. ^{1}H NMR spectra of LC copolymers. A: mPEG$_{43}$-*b*-P[(TMC-C)$_{20}$-TMC$_{20}$]; B: mPEG$_{43}$-*b*-P[(TMC-C)$_{15}$-HTMC$_5$-TMC$_{20}$]; C: mPEG$_{43}$-*b*-P[(TMC-C)$_{12}$-HTMC$_8$-TMC$_{20}$].

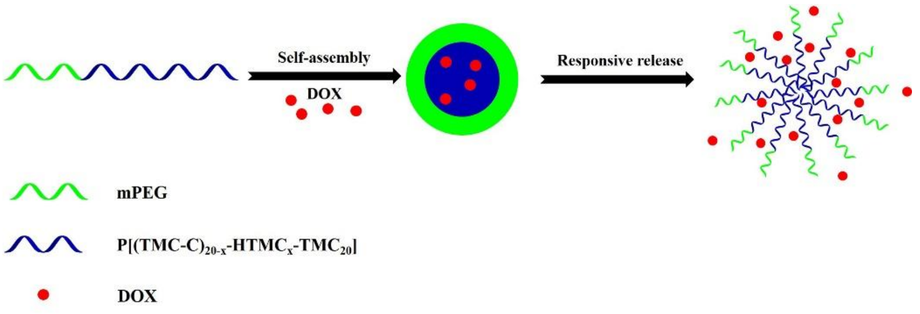

Figure S2. The simulation diagram of DOX loading and realease behavior.
